# Mesenchymal stromal cells, metabolism, and mitochondrial transfer in bone marrow normal and malignant hematopoiesis

**DOI:** 10.3389/fcell.2023.1325291

**Published:** 2023-12-18

**Authors:** Abhishek K. Singh, Parash Prasad, Jose A. Cancelas

**Affiliations:** ^1^ Division of Experimental Hematology and Cancer Biology, Cincinnati Children’s Hospital Medical Center, Cincinnati, OH, United States; ^2^ Hoxworth Blood Center, University of Cincinnati College of Medicine, Cincinnati, OH, United States

**Keywords:** bone marrow, mesenchymal stem cells, adipocytes, hematopoiesis, hematological malignancies, metabolism, mitochondrial transfer

## Abstract

Hematopoietic stem cell (HSC) transplantation-based treatments are in different phases of clinical development, ranging from current therapies to a promise in the repair and regeneration of diseased tissues and organs. Mesenchymal stromal/stem cells (MSCs), which are fibroblast-like heterogeneous progenitors with multilineage differentiation (osteogenic, chondrogenic, and adipogenic) and self-renewal potential, and exist in the bone marrow (BM), adipose, and synovium, among other tissues, represent one of the most widely used sources of stem cells in regenerative medicine. MSCs derived from bone marrow (BM-MSCs) exhibit a variety of traits, including the potential to drive HSC fate and anti-inflammatory and immunosuppressive capabilities via paracrine activities and interactions with the innate and adaptive immune systems. The role of BM-MSC-derived adipocytes is more controversial and may act as positive or negative regulators of benign or malignant hematopoiesis based on their anatomical location and functional crosstalk with surrounding cells in the BM microenvironment. This review highlights the most recent clinical and pre-clinical findings on how BM-MSCs interact with the surrounding HSCs, progenitors, and immune cells, and address some recent insights on the mechanisms that mediate MSCs and adipocyte metabolic control through a metabolic crosstalk between BM microenvironment cells and intercellular mitochondrial transfer in normal and malignant hematopoiesis.

## 1 Introduction

Stem cell transplantation-based treatments have demonstrated considerable promise in the repair and regeneration of damaged tissues and organs in a variety of degenerative disorders. Mesenchymal stromal/stem cells (MSCs), defined as non-hematopoietic, plastic-adherent, fibroblastic colony-forming cells, are among the most frequently used cell types in translational medicine. These cells are multipotent heterogeneous progenitors, able to self-renew, and differentiate into multiple cell lineages, such as chondrocytes, osteoblasts, and adipocytes. MSCs reside in the bone marrow (BM) and in a variety of other tissues and organs, including the adipose tissue, placenta, skin, fallopian tubes, cord blood, liver, and lungs (Pittenger et al., 1999). MSCs can engraft after intrafemoral administration (Abbuehl et al., 2017) but seem to be short-lived after intravenous administration due to apoptosis (Preda et al., 2021), which hampers their ability to engraft robustly in the adult BM (Poloni et al., 2006; Eggenhofer et al., 2012). Despite these limitations, numerous studies have showcased the broad anti-inflammatory and immunosuppressive properties of infused MSCs even if undergoing apoptosis (Abkowitz et al., 1992; Luk et al., 2016; Galleu et al., 2017).

MSCs, through their interaction with the innate and adaptive immune systems and tissue regeneration capacity, emerged in the last 2 decades as a promising tool in regenerative medicine and some contexts of immunosuppression (Keating, 2012; Bernardo and Fibbe, 2013). *Ex vivo* expanded human MSCs have been demonstrated to be efficacious in pre-clinical models of hematopoietic stem cell (HSC) transplantation (HSCT) and have also been employed in phase I/II clinical trials to facilitate the hematopoietic engraftment and alleviate acute graft-versus-host disease (GvHD) (Noort et al., 2002; Le Blanc et al., 2004; Bernardo and Fibbe, 2015; Burnham et al., 2020; Ringden et al., 2022). In a recent phase III clinical trial, remestemcel-L, an *ex vivo* culture-expanded allogeneic adult human MSC, has been developed as a first-line therapy in pediatric patients with steroid-refractory acute GvHD (Kurtzberg et al., 2020). *Ex vivo* expansion of cord blood-derived HSC and progenitor cells (HSPCs) cultured on BM mesenchymal lineage stroma maintains the long-term primitive HSC (CD34^+^CD38^−^) immunophenotype and improves the engraftment kinetics (Robinson et al., 2006; de Lima et al., 2012; Mehta et al., 2017).

**FIGURE 1:**
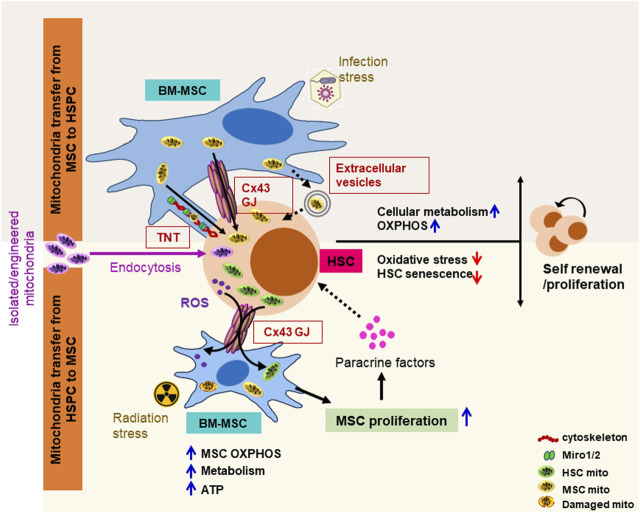
Schematic representation of mitochondria transfer mechanisms between BM-MSCs and HSCs in response to blood formation emergencies. Intercellular mitochondrial exchange between BM-MSCs and HSPCs occurs through (1) tunneling nano-tubules (TNTs) via stabilization of cytoskeleton element (F-actin, myosin, and tubulin) and membrane adapter proteins (miro1/2); (2) gap junction (GJ) channels such as connexin 43 (Cx43), where two juxtapositioned connexin hemichannels form pores connecting two neighboring cells, facilitating bidirectional mitochondrial exchange; and (3) extracellular vesicles ranging from 0.1 to 1 μm formed by blebbing of the plasma membrane. Different stress signals differentially regulate the intercellular mitochondrial exchange between MSCs and HSCs to improve the hematopoietic outcome. Following myeloablative irradiation and HSPC transplantation, transplanted HSPCs transfer functional mitochondria to BM-MSC via the gap junction protein connexin 43 in a cell contact-dependent manner, enhances MSC metabolic recovery, ATP levels, and proliferation, which, in turn, by regulating the BM niche factors (CXCL12, SCF, IL-7, osteopontin, and angiopoietin-1) improves HSPC proliferation/survival. By contrast, in infection stress hematopoiesis, BM-MSCs facilitate the transfer of mitochondria to HSPCs via TNTs and/or Cx43 GJ, improve HSPC metabolism by switching from glycolysis to oxidative phosphorylation, and subsequently stimulate hematopoietic recovery. Exogeneous-isolated functional mitochondria can be efficiently transferred into HSPCs through macropicnocytosis/endocytosis. These transferred mitochondria metabolically upgrade HSPC and boost long-term engraftment potential. ATP, adenosine triphosphate; Miro1/2, mitochondrial RhoGTPase 1/2; mito, mitochondria.

In BM, MSCs are the critical components of the HSC niche, regulating the composition of the niche environment and the hematopoietic process by secreting paracrine factors. Over the last decade, there has been a surge of interest in BM-MSC metabolism, which plays a crucial role in the regulation of surrounding niche architecture and HSCT outcomes in both normal physiology and pathological conditions. A growing body of research describes a link between MSC metabolic reprogramming via mitochondria transfer and tissue repair/regeneration (Islam et al., 2012; Mistry et al., 2019; Golan et al., 2020) (Figure 1). This mitochondrion-mediated MSC metabolic reprogramming, on the other hand, becomes poised in malignancy, modifies leukemia cell metabolism by mitochondrial transfer, and provides a survival advantage, following chemotherapy (Moschoi et al., 2016; Marlein et al., 2017; Burt et al., 2019; Forte et al., 2020; Saito et al., 2021). This review highlights new concepts on the mechanisms through which BM-MSCs interact with the surrounding HSPCs and address the mechanism that mediates MSC metabolic reprogramming to provide a better understanding of MSC-based therapies in normal and malignant hematopoiesis.

## 2 Identity and characterization of BM-MSCs

BM-MSCs are often perivascular, wrapped tightly around arterioles and more loosely around the sinusoidal blood vessels (Crisan et al., 2008; Crane et al., 2017; Wei and Frenette, 2018; Pinho and Frenette, 2019). The varied nature of the MSC population and the requirement to identify functionally distinct subgroups present a challenge to understanding the function of MSCs in the HSC niche. In human tissue, MSCs are recognized based on the presence of CD44, CD90, CD105, CD73, and CD106 and the lack of hematopoietic and endothelial (CD31) cell markers (Dominici et al., 2006; Schwab and Gargett, 2007; Crisan et al., 2008; Ouzin and Kogler, 2023). However, the MSCs isolated from different sources exhibit differential gene expression patterns, and over the years, a significant number of markers have been proposed to aid in the isolation of MSCs from their surroundings. BM-MSCs can be enriched using a combination of stemness markers such as Stro-1, SSEA-4, CD271, and CD146 (Buhring et al., 2007; Lv et al., 2014). MSCs expressing CD146 persist around the sinusoidal blood vessels in the ossicles, highly express the HSC niche factor angiopoietin-1, and regenerate bone and stroma to establish a hematopoietic milieu and favor HSPC proliferation (Sacchetti et al., 2007; Schwab and Gargett, 2007; Tormin et al., 2011). In contrast, CD271^+^ MSC localized in the trabecular region of human BM associated with the maintenance of long-term HSPCs in low-oxygen areas. These cells lack the ability to express CD90 and CD73, and when combined with minimal or no expression of PDGFRα, define the MSC subgroup promoting *ex vivo* expansion of transplantable CD34^+^ HSC (Tormin et al., 2011; Li et al., 2014; Crippa et al., 2021). Both CD271^+^CD146^−/low^ and CD271^+^CD146^+^ cells were observed to generate colony-forming unit–fibroblasts (CFU-Fs) *in vitro*. However, MSCs could only self-renew in serial transplantation *in vivo* when grown as tridimensional mesenspheres and not as plastic-adherent CFU-Fs, demonstrating that plastic-adherent CFU-Fs contain very few primitive MSCs (Ghazanfari et al., 2016). In a recent study, Zhang et al. (2023), by performing a comprehensive screening of human fetal BM-nucleated cells at single-cell resolution, discovered LIFR^+^PDGFRB^+^CD45^−^CD31^−^CD235a^−^ (LIFR^+^PDGFRB^+^) and TM4SF1^+^CD44^+^CD73^+^CD45^−^CD31^−^CD235a^−^ (TM4SF1^+^CD44^+^CD73^+^) mesenchymal progenitors. By performing *in vivo* transplantation, they showed that only the LIFR^+^PDGFRB^+^ MSC subset can effectively restore the hematopoietic microenvironment *in vivo*, whereas TM4SF1^+^CD44^+^CD73^+^ cells are preferentially committed to osteogenic differentiation (Zhang et al., 2023). RNA velocity and cell–cell communication analysis further identified six transcriptionally and functionally distinct stromal cell populations, demonstrating that CD45^low/−^CD235a^−^CD71^−^CD271^+^NCAM1^−^CD52^−^CD81^++^ MSCs have the highest CFU-F potential and tri-lineage differentiation capacity and interact with essentially all hematopoietic cell types through CXCL12 pathways (Li et al., 2023).

Mouse BM-MSCs are identified by the presence of PDGFRα, CD29, CD49e, CD44, CD73, CD105, and Sca-1, with the absence of hematopoietic and endothelial cell markers (Morikawa et al., 2009; Breitbach et al., 2018). Self-renewing mouse MSCs are highly concentrated in the PDGFRα^+^Sca-1^+^CD45^−^Ter119^−^ (PαS) population, preferentially reside in the arterial perivascular space near the inner surface of the cortical bone, and differentiate into osteogenic, chondrogenic, and adipogenic populations. These cells selectively express HSC niche factor angiopoietin-1. On the other hand, the CXCL12-abundant reticular (CAR) cells, phenotypically identified as PDGFRα^+^Sca-1^−^CD45^-^Ter119^−^ MSCs, primarily found surrounding sinusoids induce adipo-osteogenic bipotential progenitors and express high levels of CXCL12 to maintain hematopoiesis (Morikawa et al., 2009; Omatsu et al., 2010). Lineage tracing and fate mapping studies further identified nestin and leptin receptor (leptinR^+^), expressing perivascular MSCs in BM (Mendez-Ferrer et al., 2010; Ding et al., 2012; Greenbaum et al., 2013; Kunisaki et al., 2013; Zhou et al., 2014; Wei and Frenette, 2018; Pinho and Frenette, 2019; Severe et al., 2019; Kara et al., 2023). Nestin^+^ cells have been shown to originate from the neural crest and together with an increased capacity to differentiate into mesodermal cells, release of colony-stimulating factor-1 (CSF-1; OR macrophage-stimulating-factor; M-CSF), and the tissue inhibitors of metalloproteinase (TIMP)-1 and -2 support the HSC function (Mendez-Ferrer et al., 2010; Kunisaki et al., 2013). LeptinR^+^ cells are heterogenous, with peri-arteriolar leptinR^+^ cells poised to undergo osteogenic differentiation, while peri-sinusoidal leptinR^+^ cells poised to undergo adipogenic differentiation and support hematopoiesis *in vivo* by secreting several niche factors, such as CXCL12, SCF, and angiopoietin-1 (Zhou et al., 2014; Shen et al., 2021). LeptinR^+^CD45^-^Ter119^−^ represent 0.2%–0.3% of enzymatically dissociated BM cells and are highly overlapping with PDGFRα^+^CD45^−^Ter119^−^CD31^−^ MSCs. Additionally, MSC markers CD51 and PDGFRβ are consistently expressed by these LepR^+^CD45^−^Ter119^−^ cells (Zhou et al., 2014; Shen et al., 2021). Contrarily, nestin-GFP^+^CD45^−^Ter119^−^CD31^−^ MSCs represent a small subset (0.08%) of BM-nucleated cells and uniformly express PDGFRα and CD51. Other mesenchymal lineage markers were either heterogeneously expressed (CD29, CD44, CD61, CD130, and P75) or restricted to a small subset of nestin^+^ cells (CD10, CD90, CD166, and CD133) (Mendez-Ferrer et al., 2010; Pinho et al., 2013). Nestin transgenes display different expression patterns in BM, and by using reporter and conditional gene-targeted mice, Kunisaki et al., identified a rare (0.002% of total BM) sympathetic nervous system-innervated nestin-GFP^bright^ subset that is positive for the pericyte marker NG2 (chondroitin sulfate proteoglycan-4, Cspg4) and α-smooth muscle actin (α-SMA) and regulates HSC quiescence (Kunisaki et al., 2013). In a recent study, Kara et al. (2023), employing scRNA seq analysis, showed that leptinR^+^ MSCs are the primary sources of SCF and CXCL12 in early postnatal and adult BM. Intriguingly, within nestin^+^ MSCs, SCF and CXCL12 clusters only coincide with nestin^+^SMA^+^ cells (Kara et al., 2023).

Single-cell protein expression mapping has demonstrated that leptinR^+^ and nestin^+^ BM-MSCs are highly susceptible to myeloablative conditioning, and the stromal cell subset defined as CD105^−^CD73^+^NGFR^high^ participates in HSPC engraftment and initial stages of hematological recovery post-irradiation (Severe et al., 2019). Of note, the radio-tolerant CD105^−^CD73^+^NGFR^high^ stromal cells express a high level of CXCL12 with minimal or no overlap with leptinR^+^ and nestin^+^ MSCs (Severe et al., 2019). Further studies employing *in vivo* ectopic bone-forming assay and differential expression of cell surface markers revealed MSC hierarchy in BM, where CD45^−^Ter119^−^CD31^−^CD166^−^CD146^−^Sca1^+^ (Sca1^+^) cells are most primitive, giving rise to CD45^−^Ter119^−^CD31^−^CD166^−^CD146^+^ (CD146^+^) intermediate progenitors and mature CD45^−^Ter119^−^CD31^−^CD166^+^CD146^−^(CD166^+^) osteo-progenitors (Hu et al., 2016). All these mesenchymal progenitors preserve *in vitro* HSC long-term multi-lineage reconstitution potential. However, their differentiation potential differs dramatically *in vivo*, and only most primitive Sca1^+^ mesenchymal stromal progenitors, which coincide with leptin receptor-expressing MSCs, differentiate into CXCL12-producing stromal cells to support hematopoiesis (Hu et al., 2016). Considering the fact that MSCs are highly heterogenous and differentially regulate HSC composition, the *ex vivo* expansion of MSCs on the plastic surface induces phenotypic and functional changes, and myeloablative conditioning differentially impairs BM-resident MSC fate and function, further studies are required to identify the clinically relevant MSC subsets to improve the hematopoietic outcome.

## 3 Role of BM-MSC and its adipocytic lineage in hematopoiesis

Inadequate engraftment of HSCs is a major cause of morbidity after clinical HSCT. Notwithstanding, myelosuppression, which lasts for several weeks in post-transplant patients, deteriorates the adaptive immunity and raises the risk of opportunistic infections (Storek et al., 2008). Although multiple studies have focused on understanding the HSC biology that sustains native hematopoiesis, therapeutic strategies promoting BM niche reconstitution after myelosuppressive injury are essential to improve clinical HSCT outcomes. Multiple BM niche cells differentially support and preserve the integrity of hematopoiesis. MSCs constitute an essential component of the BM niche and is one of the main regulators of HSPC homeostasis by secreting paracrine factors like CXCL12, SCF, IL-7, osteopontin, angiopoietin-1, and VCAM-1, as well as by establishing a cell-to-cell contact (Hooper et al., 2009; Butler et al., 2010; Schajnovitz et al., 2011; Ding et al., 2012; Mendelson and Frenette, 2014; Ziegler et al., 2016; Guo et al., 2017; Himburg et al., 2018; Chen et al., 2019; Batsivari et al., 2020; Singh and Cancelas, 2020; Yin et al., 2020). The potent immunomodulatory properties of MSCs offer therapeutic benefits in the management and prevention of GvHD and in the promotion of tissue regeneration and engraftment, following HSCT.

BM adipocytes (BMATs) play opposing roles in the process of hematopoiesis. Different cytokines, including CXCL12, IL-8, CSF3, and LIF, which positively control HSC survival and provide essential niche factors, are generated from BM adipocytes (Mattiucci et al., 2018; Wilson et al., 2018). These cells also produce several hematopoietic supportive factors and regulate HSC homing. In contrast, others observed that BM adipocytes function as a negative regulator of hematopoiesis (Naveiras et al., 2009; Zhu et al., 2013; Lu et al., 2016), and depending on the anatomic location (proximal-regulated-rBMAT vs. distal-constitutive-cBMAT) within the BM, they act differently (Tratwal et al., 2021). In comparison to cBMATs, rBMATs may be the primary regulator of hematopoiesis; however, further research with appropriate origin information on these different types of BMATs is needed to justify their involvement in hematopoiesis.

### 3.1 Mesenchymal stem cells facilitate hematopoietic engraftment

HSC mainly resides in the specialized niche of the BM, and myeloablative preconditioning for HSCT by total body irradiation or chemotherapy not only depletes the recipient’s HSCs but also permanently incapacitates the sinusoidal blood vessels and BM stromal cells (Abbuehl et al., 2017; Tikhonova et al., 2019). In the past decade, several preclinical and clinical investigations have shown that co-transplantation of MSCs with HSCs facilitates the migration and homing of the HSCs to recipient BM niches and improves hematopoietic recovery (Noort et al., 2002; Maitra et al., 2004; Muguruma et al., 2006; Le Blanc et al., 2007; Abbuehl et al., 2017; Tikhonova et al., 2019; Zhang et al., 2023). Transplantation of adult BM cells from EGFP transgenic animals can generate a population of Lin^−^/Sca-1^+^/c-kit^−^ BM cells, albeit at low levels, in the BM of osteogenesis imperfecta (OI) recipients when transplanted intra-utero (Panaroni et al., 2009). Unfortunately, this study did not report engraftment of purified, fresh, or expanded MSCs into the same OI mice. Transplantation of a freshly isolated primary BM-MSC demonstrated that only a rare CD73^+^/CD105^−^/Sca1^+^ population within the BM-MSCs engrafts long-term, possesses self-renewal potential, and regenerates multilineage BM niche cells to support hematopoiesis, when co-transplanted intrafemorally with HSC (Abbuehl et al., 2017). Furthermore, following 3D whole-mount imaging by light-sheet microscopy, they observed that the engrafted MSC primarily localizes to the metaphysis and cortical region of bone close to HSCs (Abbuehl et al., 2017).

The current view is that infused BM-MSCs are, in general, short-lived (Poloni et al., 2006; Eggenhofer et al., 2012; Preda et al., 2021). However, a non-apoptotic subpopulation of BM-MSCs may survive and remain dormant during homeostasis. In response to stress/injury, these MSCs may proliferate and differentiate to support emergency hematopoiesis. Epidermal growth factor (EGF), fibroblast growth factor-2 (FGF-2), and PDGFβ are amongst the BM environment-derived cytokines shown to promote long-term *ex vivo* expansion and differentiation of MSCs (Ng et al., 2008; Tamama et al., 2010; Itkin et al., 2012; Doan et al., 2013; Chen et al., 2015; Han et al., 2022). Of note, MSCs engineered to overexpress PDGFβ exhibit improved survival and growth after transplantation and dramatically promote the engraftment of the human HSC in immunodeficient mice (Yin et al., 2020). Due to the limited availability of human tissues, MSCs must be expanded *ex vivo* to meet the cellular demand and transplantable HSPC expansion. A large cohort of studies have suggested that following the *ex vivo* culture, BM-MSCs gradually lose their proliferative and secretory capabilities and alter the expression of key regulators of HSC self-renewal and maintenance (Abbuehl et al., 2017; Nakahara et al., 2019; Zhang et al., 2023). The expression of Spp1 (osteopontin), a negative regulatory element of the BM niche that limits the size of the stem cell pool, is substantially upregulated (Abbuehl et al., 2017), while the expression of early growth response 1 (Erg1) and nestin was substantially downregulated (Nakahara et al., 2019; Li et al., 2020), which is correlated with the loss of proliferative and secretory activities of the cultured MSCs. The genetic alteration in BM niche cells leads to long-term functional changes in BM HSCs as overexpression of the transcription factor EGR1 in BM-MSCs improves the hematological stroma support via CCL28 and VCAM1 induction, and promoted the *ex vivo* expansion of transplantable CD34^+^CD90^+^ HSC (Li et al., 2020). Nakahara et al. (2019), following RNA sequencing screen, identified five genes encoding transcription factors (Klf7, Ostf1, Xbp1, Irf3, and Irf7) that fully restored the HSC niche function in the cultured BM-derived MSCs. Notably, the expression of the PαS cell-specific early B-cell factor (EBF) family of transcription factors, specifically Ebf1 and Ebf3, is essential for the preservation of HSCs and myeloid/lymphoid lineage output, and exhibits the memory phenotype that endures post-transplants (Derecka et al., 2020; Nakatani et al., 2023).

Clinically, MSC infusions are, in general, safe, with no adverse effects (Gao et al., 2016). Co-transplantation of BM-MSCs in the context of cord blood HSC transplantation accelerates short-term hematopoietic engraftment (de Lima et al., 2012). A positive impact of the MSC co-transplant has also been observed in lymphoma and myeloma patients with an increased production in memory and naïve T cells (Batorov et al., 2015) and reducing GvHD after allogeneic HSCT (Burnham et al., 2020; Macías-Sánchez et al., 2022), possibly through cytokine-mediated promotion of CD5^+^ B-cell production and downregulation of NK cells, CD4^+^ T cells, and/or macrophages (Lin et al., 2023). MSCs can also compromise the immune cell activity in leukemia patients, which may associate with disease recurrence after HSCT and MSC co-transplantation (Ning et al., 2008).

### 3.2 MSC mitochondrial transfer in normal hematopoiesis

BM niche cells form a heterogeneous population of cells that, upon stress activation, can exhibit specific single-cell responses which differentially affect the hematopoietic outcomes of surrounding HSPCs (Vannini et al., 2016; Vannini et al., 2019; Hinge et al., 2020; Liang et al., 2020). The crosstalk between the HSPC and BM microenvironment is critical for homeostasis and hematopoietic regeneration in response to blood formation emergencies. BM niche cells undergo massive damage to their mitochondrial function after myeloablative irradiation and/or pathogen infection, which eventually impairs hematopoietic regeneration (Figure 1). HSCs are exquisitely sensitive to irradiation, and even a low dose of ionizing radiation can increase the levels of reactive oxygen species (ROS) enough to activate p38MAPK-dependent senescence and apoptosis programs or even just impair their self-renewal capacity (Henry et al., 2020). LeptinR^+^ and nestin^+^ stromal cell subsets are also irradiation-sensitive, albeit at a much lesser degree (Severe et al., 2019), where moderate ROS levels are tolerated and even exert positive effects (Sheppard et al., 2022). At higher levels, ROS accumulation associated with inflammatory stress through the alteration of the RIG-I-Trim25-Keap1-NRF2 complex, which impairs the clonogenic capacity and bone-forming ability of BM-MSCs (Lou et al., 2022).

Intercellular mitochondrial transport, which refers to the transfer of either mitochondrial DNA or the entire organelle to supplement cellular energy demand in metabolically compromised recipient cells, has been documented both *in vitro* and *in vivo* in a variety of cells under physiological and pathophysiological conditions (Moschoi et al., 2016; Chandran et al., 2017; Marlein et al., 2017; Burt et al., 2019; Golan et al., 2020; Shanmughapriya et al., 2020; Brestoff et al., 2021; Saito et al., 2021; Borcherding et al., 2022). This intercellular mitochondrial transfer occurs through different means like intercellular tunneling nanotube (TnT) bridges or secreted small vesicles and frequently requires connexin 43 (Cx43)-containing gap junction (GJ) channels (Qin et al., 2021; Singh and Cancelas, 2021) (Figure 1).

Our previous research has demonstrated that hematopoietic regeneration and efficient blood formation after myeloablation depend on the transfer of damaging ROS from HSPC to the BM microenvironment, and hematopoietic Cx43 facilitates this transfer and prevents ROS-p38MAPK-p16/INK4a-mediated HSC senescence (Taniguchi Ishikawa et al., 2012) (Figure 1). Mitochondria are the major source of cellular bioenergetics and an important regulator of HSC fate decisions and energy homeostasis (Ito et al., 2004; Liu et al., 2015; Ito et al., 2016; Khacho et al., 2016; Luchsinger et al., 2016; Hu et al., 2018; Jin et al., 2018; Umemoto et al., 2018; Hinge et al., 2020). Under steady-state conditions, HSCs are primarily quiescent, rely on anaerobic glycolysis, and exhibit low mitochondrial ROS and mitochondrial membrane potential. However, upon regenerative stress, as found after myeloablative or infection stress, HSC undergoes rapid division and switches to mitochondrial oxidative phosphorylation to meet the energy demands (Ito et al., 2004; Maryanovich et al., 2015; Ito et al., 2016; Khacho et al., 2016; Luchsinger et al., 2016; Hu et al., 2018; Umemoto et al., 2018). Our group identified a new approach of hematopoietic recovery post-conditioning and demonstrated that following whole-body irradiation and HSPC transplantation, transplanted HSPCs through the transfer of mitochondria to the irradiated host MSC improve the metabolic recovery and proliferation of recipient BM stromal cells, which, in turn, increases the hematopoietic reconstitution (Golan et al., 2020) (Figure 1). We further identified that expression of Cx43 and low adenosine 5′-monophosphate-activated protein kinase (AMPK) activity in the HSPC compartment is crucial for rapid hematopoietic reconstitution after transplantation (Golan et al., 2020).

Furthermore, there is much evidence that BM-MSCs transfer mitochondria to HSPC to resist the oxidative stress via metabolic reprogramming and improve the hematopoietic reconstitution (Marlein et al., 2017; Mistry et al., 2019) (Figure 1). In infection stress hematopoiesis, BM-MSCs facilitate the transfer of mitochondria to HSPCs, which improves cellular metabolism by switching from glycolysis to oxidative phosphorylation and subsequently stimulates granulopoiesis (Mistry et al., 2019). This study further investigated the BM microenvironment heterogeneity in terms of mitochondria transfer and demonstrated that in response to infection, ROS-induced oxidative stress promotes phosp-protein kinase B (AKT) signaling, which drives Cx43 channel-mediated mitochondria transfer from BM-MSCs but not from other niche cells to HSPCs (Mistry et al., 2019). Recently, Jacoby et al. (2021), following *ex vivo* supplementation of functional mitochondria in human cord blood CD34^+^ cells, showed that mitochondrial augmentation boosts HSPC bioenergetics, enables T-cell expansion, and improves long-term engraftment in the NSGS mice (Figure 1). Although HSPCs and preclinical models highlight the beneficial effect of HSPC and BM-MSC mitochondrial exchange and the cellular bioenergetic shift in emergency hematopoiesis, the potential therapeutic implications of mitochondrial transfer in steady-state and stress hematopoiesis, the specific interaction between the HSPC and BM stromal cells, and mechanisms regulating intercellular mitochondrial exchange require further investigation.

## 4 Role of MSCs and their adipocytic lineage in hematological malignancies

Despite the therapeutic benefit of BM-MSCs, it has become poised in malignancy, and it has been observed that in hematological malignancies, remodeling of the BM niche creates an environment that favors malignant stem cells and hinders normal hematopoiesis (Forte et al., 2020). Additionally, through direct cell-to-cell contacts that frequently change the metabolism of cancer cells, dysregulation of extracellular matrix deposition, and modification of soluble factors/metabolites, BM-MSCs offer a survival advantage post-chemotherapy. Substantial evidence suggests that acute myelogenous leukemia (AML)-associated BM-MSCs show reduced CXCL12 and SCF expression with reduced sinusoidal and increased arterial cell populations (Arranz et al., 2014; Baryawno et al., 2019). Furthermore, an imbalanced BM-MSC/endothelial cell ratio in B-cell precursor acute lymphoblastic leukemia (BCP-ALL) post-therapy associated with a shorter disease-free survival, irrespective of minimal residual disease status (Oliveira et al., 2022). Likewise, myelodysplastic syndrome (MDS)-driving mutations require the presence of altered stromal cells and MSCs in an aged milieu, where they express senescence markers, elevated inflammatory molecules, and reduced levels of cytokines required for normal hematopoiesis (Huang et al., 2015; Fernando et al., 2019; Plakhova et al., 2023).

Myeloproliferative neoplasms have also been linked to increased BM-MSC apoptosis (Arranz et al., 2014; Corradi et al., 2018; Genitsari et al., 2018). Sympathetic nerve cells maintain BM Schwann cells and nestin^+^ cells, whereas leukemia progression is facilitated by a reduction in nerve fibers, which results in the apoptosis of nestin^+^ MSCs and a subsequent drop in CXCL12 levels. Treatment with β3-adrenergic agonists effectively restores the BM-MSC pool, followed by leukemia inhibition (Arranz et al., 2014). There are no notable alterations to the cytomorphology of BM-MSC or marker-based discrepancies during the progression of AML or B-ALL however, a substantial and varied change in the gene/protein expression patterns are observed, resulting in decreased cell proliferation and CFU-F-forming ability similar to that of senescent BM-MSCs (Le et al., 2016; Desbourdes et al., 2017; Genitsari et al., 2018; Baryawno et al., 2019; Fernando et al., 2019; Zhang et al., 2021).

**FIGURE 2:**
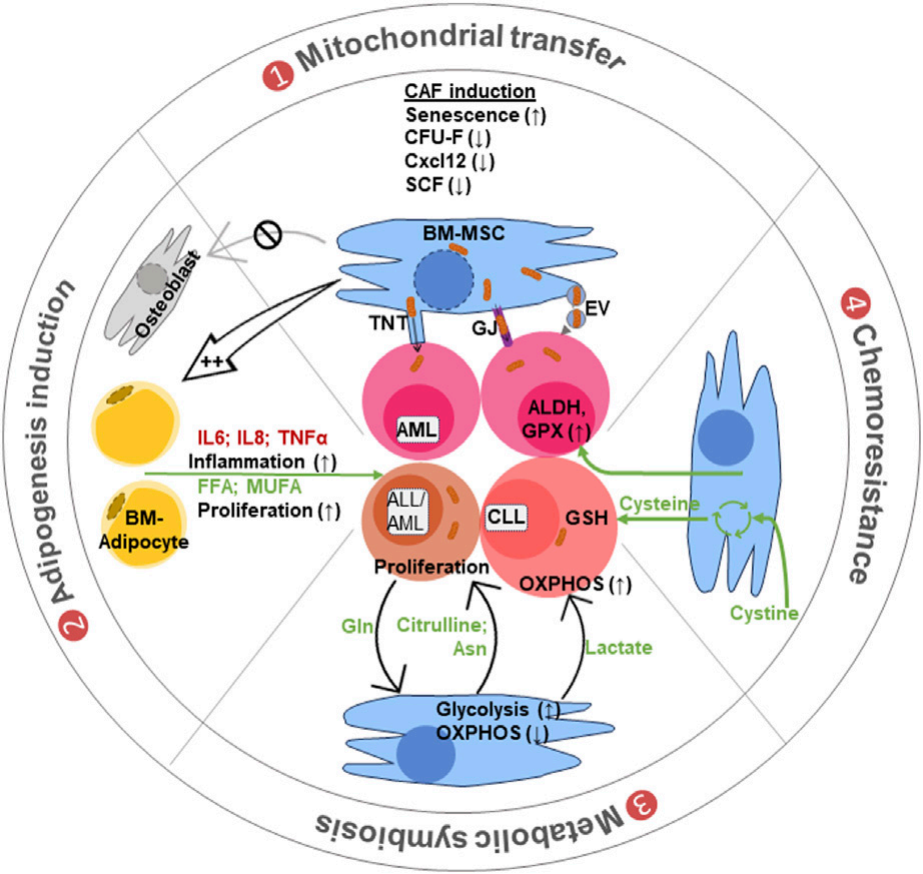
Different modes of BM-MSC crosstalk with leukemic cells. (1) Mitochondrial transfer: leukemic cell interaction converts the BM-MSCs into cancer-associated fibroblasts (CAFs) with altered features to support leukemia. Mitochondrial transfer from MSCs to leukemic cells occurs through tunneling nanotubes (TnTs), gap junctions (GJs), or via mitochondria carrying extracellular vesicles (EVs). (2) Adipogenic induction: these CAFs are biased toward adipocyte production with reduced osteoblast differentiation. Adipocytes support leukemia progression by secreting inflammatory cytokines and lipid molecules like free fatty acid (FFA) and monounsaturated fatty acids (MUFA) for cell growth and proliferation. (3) Metabolic symbiosis: the BM stromal cells supply different metabolites like lactate to maintain OXPHOS and amino acids like asparagine and glutamine (also citrulline), for biosynthetic needs of leukemic cells. (4) Chemoresistance: BM-MSCs also provide chemoresistance by providing cysteine for glutathione (GSH) synthesis. They also induce aldehyde dehydrogenase (ALDH) and glutathione peroxidase (GPX) in leukemic cells to metabolize chemotherapeutic drugs and reduce their effects.

Metabolically, proliferating transformed leukemic cells also consume glutamine and asparagine from their surrounding BM niches (Samudio and Konopleva, 2013; Bolzoni et al., 2016; Jiang et al., 2021). As glutamine and, to a lesser extent, asparagine are important for BM-MSC proliferation, lineage allocation, stemness, and osteogenesis (Zhou et al., 2019; Zhou et al., 2019; Sharma et al., 2021; Lungu et al., 2023), extracellular depletion of glutamine and asparagine, by proliferating leukemic cells, may impair MSC functionality and induce MSC senescence and osteopenia (Fernando et al., 2019; Gooding et al., 2019) (Figure 2).

### 4.1 BM-MSC-derived adipocytes positively regulate proliferation and survival of hematological malignant cells

BM stromal cells, especially adipocytes, are the source of different inflammatory molecules like IL-6, IL-8, IL-1B, IL-15, IL-17, and TNF-α; they can support clonal hematopoiesis and leukemia progression (Figure 2). The majority of studies have shown a positive correlation between adipogenicity with reduced osteogenic potential of BM-MSC and BM leukemia progression (Le et al., 2016; Fernando et al., 2019; Azadniv et al., 2020; Weickert et al., 2021; Kobari et al., 2022). The co-culture of mixed lineage leukemia (MLL) with BM-MSC results in downregulation of the expression of the early adipogenic cell fate inhibitor DLK1 in BM-MSCs (Weickert et al., 2021). Likewise, patients with multiple myeloma (MM) display higher levels of cytokines (ANG1, ENA-78, EGF, PDGF-AA/AB/BB, and TARC) that were linked to decreased osteoblastic differentiation and increased expression of CD36 and PPARγ with a skewing toward adipogenesis (Kobari et al., 2022). In contrast, Boyd et al. (2017), by performing *in vitro* co-culture and *in vivo* xenograft modeling, revealed that leukemia cells disrupt the adipocytic niche in BM, which led to the imbalanced regulation of endogenous HSPCs and hamper myelo-erythroid maturation. An *in vivo* administration of PPARγ agonists increases the BM adipocyte content and rescues healthy hematopoiesis (Boyd et al., 2017).

Proliferating leukemic cells consume large amounts of glucose (Warburg et al., 1927) for biosynthetic material and adenosine-5′-triphosphate (ATP) production, exceeding the biosynthetic needs (Vander Heiden et al., 2009). Continuous enhanced glycolysis faces a limitation of nicotine adenine dinucleotide, oxidized (NAD^+^). A sustained high rate of glycolysis results in rapid consumption of NAD^+^, which obliges tumor cells to consume extracellular pyruvate. To recover pyruvate, tumor cells use alternative fuels including fatty acids, glutamine, asparagine, serine, or aspartate (Sullivan et al., 2018; Li et al., 2022). Adipocytes represented the most important source of BM mono-unsaturated fatty acids, a preferred substrate for cancer cell membrane synthesis, which are less prone to fatty acid oxidation and ferroptosis than poly-unsaturated fatty acids (Hargrove et al., 2001; Gan, 2021; Mao et al., 2021). The differential effect of the BM microenvironment on leukemia metabolism is evident in B-cell acute lymphoblastic leukemia (B-ALL). The expression of stearoyl-coenzymeA-desaturase in BM B-ALL cells is reduced, making leukemic cells dependent on BM adipocytes to supply the mono-unsaturated free fatty acids for their survival and proliferation (Savino et al., 2020), illustrating the metabolic coupling of leukemic cells with the BM microenvironment.

### 4.2 BM-MSCs in chemotherapy resistance

BM-MSCs provide chemoresistance against retinoic acid (R)^+^, the proteasome inhibitor bortezomib (B), and the oxidative stress inducer arsenic trioxide (A) RBA (Liccardo et al., 2023). Nestin^+^ BM-MSCs support survival and chemotherapy relapse of AML through increased oxidative phosphorylation, tricarboxylic acid (TCA) cycle activity, and glutathione (GSH)-mediated antioxidant defense (Forte et al., 2020) with increased GPX activity. Interestingly, some reports suggest that the inflamed BM stromal cell microenvironment may increase the vulnerability of AML toward chemotherapies (Chen et al., 2023; Lisi-Vega and Méndez-Ferrer, 2023).

Zhang et al. (2012) demonstrated that chronic lymphocytic leukemia (CLL) cells express very low levels of the cysteine/glutamate antiporter xCT, preventing them from uptaking extracellular cystine for GSH synthesis. BM-MSCs actively uptake cystine and release cysteine for CLL uptake, thus helping in GSH synthesis and protection against ROS and drug-induced cytotoxicity (Figure 2). xCT inhibitors like (S)-4-carboxyphenylglycine and sulfasalazine induced chemotherapy sensitivity and cell death in CLLs. The anti-CD44 monoclonal antibody induces apoptosis and/or differentiation of AML cells via PI3K/AKT-p27 pathway upregulation in culture systems. Interestingly, BM stromal cells provide chemoresistance against anti-CD44 therapy by downregulating the AKT pathway in AML cells (Chen et al., 2015). Additionally, BM-MSCs activate the ALDH enzyme, which, through the TGFb-p38-ALDH pathway, converts xenobiotic aldehydes into less toxic carboxylic acid that is essential for drug metabolism and chemoresistance in AML (Sládek, 2003; Januchowski et al., 2013; Yuan et al., 2020) (Figure 2).

### 4.3 Metabolic crosstalk between leukemic cells and BM-MSCs

A stromal cell interaction with CLL causes a shift from mitochondrial to glycolysis-dependent bioenergetics through the NOTCH-cMYC signaling pathway that incurs increased proliferation and chemoresistance properties in CLL cells (Koppenol et al., 2011; Jitschin et al., 2015). Leukemic cells (ALL and BCP-ALL) secrete leukemic extracellular vesicles (EVs) that are consumed by the BM-MSCs (Johnson et al., 2016). Following EV ingestion, these BM-MSCs exhibit increased lactate generation, glycolysis, and mitochondrial metabolism. Similar improvements in BM-MSC glycolysis and mitochondrial metabolism by the CLL secretome improve stromal cell fitness, which aids in tumor cell survival (Lazarian et al., 2022). Vangapandu et al. (2017) have shown that the oxidative phosphorylation (OXPHOS) and ribonucleotide synthesis pathway are elevated in patient-derived CLL cells. The increase in OXPHOS and ribonucleotide was successfully decreased by the small-molecule complex I inhibitor IACS-010759; nevertheless, as a compensatory strategy, glycolysis increased. Concurrent administration of 2-deoxy-glucose and IACS-010759 suppressed this compensatory glycolysis and induced CLL cells apoptosis (Vangapandu et al., 2018). Protein kinase C-β (PKCβ) also plays a critical role in the BM stroma and CLL cell interaction, where they provide a cancer-supportive inflammatory microenvironment (vom Stein et al., 2023), chemoresistance (Amigo-Jiménez et al., 2015), and establish metabolic symbiosis (von Heydebrand et al., 2021). An interaction through PKCβ reduced glycolysis and insulin signaling in the BM-stromal cells and simultaneously increased the glycolysis/lactate production in CLL cells. It has been proposed that an elevated lactate environment aids in immunosuppression (Chen et al., 2022), and the leukemic cells can utilize it to run the TCA cycle or *vice versa* (Saulle et al., 2021). Extracellular lactate binds to the lactate receptor GPCR81 and takes part in the cellular metabolism after being taken up through MCT4 channels. Furthermore, intracellular lactate-mediated histone-lysine lactylation and protein lactylation are crucial for lactate-mediated signaling (Chen et al., 2022; Liu et al., 2022; Zhang et al., 2023). Thus, targeting lactate consumption is also helpful in reducing cell proliferation and leukemic burden (Yu et al., 2020; Saulle et al., 2021; Wang et al., 2021) (Figure 2).

L-asparaginase therapy achieves approximately 90% remission rate in B-ALL when combined with doxorubicin, vincristine, and prednisone (Parmentier et al., 2015; Tabe et al., 2019; Wang et al., 2023). BM-MSCs contribute to B-ALL resistance against L-asparaginase therapy by supplying asparagine to leukemic cells (Iwamoto et al., 2007). L-asparaginase-treated leukemic blasts synthesize glutamine to be uptaken by BM-MSCs, and in return, BM-MSCs convert glutamine into asparagine that cycles back to leukemic cells (Chiu et al., 2021). BM adipocytes can also secrete glutamine and asparagine to prevent leukemic cell death by L-asparaginase, which may be at the root of the poor response of obese patients to conventional L-asparaginase containing B-ALL therapy (Samudio and Konopleva, 2013) (Figure 2).

T-ALL cells are specifically dependent on arginine levels and consume arginine produced by the BM microenvironment (Chiu et al., 2021). Based on this fact, the PEGylated arginase (pegargiminase) has been used for clinical trials (De Santo et al., 2018). However, some studies also show that BM-MSCs synthesize and secrete citrulline, which is consumed by T-ALL cells to gain chemoresistance against pegargiminase (Sugimura et al., 1990; Kwong-Lam and Chi-Fung, 2013).

Finally, extracellular glutamine is specifically utilized by AML, MM, and CLL cells for anaplerosis and amino acid production. It also aids in mTOR activation, permitting enhanced protein synthesis and leukemia burden (Samudio and Konopleva, 2013; Bolzoni et al., 2016; Chiu et al., 2021) (Figure 2).

### 4.4 BM mitochondrial transfer and leukemic chemoresistance

AML cells prefer OXPHOS and, in many situations, fatty acid oxidation (FAO) over glycolysis (Tabe and Konopleva, 2023). High aerobic glycolysis or “Warburg effect” in AML is related to better treatment efficacy and increased lifespan (Herst et al., 2011). In contrast, increased OXPHOS, mitochondrial mass, ROS, and FAO are associated with greater chemoresistance against medications such as cytarabine in AML (Moschoi et al., 2016; Farge et al., 2017; de Beauchamp et al., 2022). Conventional chemotherapy-resistant leukemic cells typically rely on a very active mitochondrial metabolism (de Beauchamp et al., 2022). These resistant cells modify their surrounding BM niches and reprogram them into cancer-associated fibroblasts (CAFs), which, in turn, help the leukemic cells by transferring functional mitochondria via tunneling nanotubes (Burt et al., 2019). Nonetheless, inhibiting CAF production with corticosteroids, intercellular communication with vincristine, and ROS detoxification with N-acetyl cysteine, glutathione, and diphenyleneiodonium selectively reduced the leukemia burden and improve survival (Burt et al., 2019).

A growing body of data indicate that leukemia cell metabolism is especially increased by mitochondrial donation from BM-MSCs, which also increases the likelihood of leukemia cell survival after chemotherapy (Moschoi et al., 2016; Marlein et al., 2017; Burt et al., 2019; Forte et al., 2020; Saito et al., 2021). The increased frequency of nestin^+^ MSCs in AML patients’ BM and in ALL murine xenograft models represents a cell-to-cell contact-dependent ROS-detoxifying mechanism that permits mitochondrial transfer to promote chemoresistance and recurrence (Wang et al., 2018; Burt et al., 2019; Moore et al., 2019) (Figure 2). In response to chemotherapy, such as daunorubicin or cytarabine treatment, which caused ROS and damaged mitochondria accumulation, formation of TnT in B-ALL patients provides a survival advantage through mitochondrial transfer from BM-MSCs. Inhibiting TnT formation with vincristine, however, limits mitochondrial transport from BM-MSCs and sensitizes leukemic cells against chemotherapy (Burt et al., 2019). Similarly, healthy mitochondria are acquired by AML and MM cells from BM-MSCs via TnT, and this acquisition is further improved, following chemotherapy (Moschoi et al., 2016; Matula et al., 2021; Saito et al., 2021). Following transfer of mitochondria, AML cell-derived NADPH oxidase 2 increases ROS accumulation in the surrounding BM-MSCs and activates their mitochondrial biogenesis through the peroxisome proliferator-activated receptor-gamma coactivator-1 alpha (PGC1α). These extra mitochondria are transferred from BM-MSCs to AML cells, trigger compensatory activation of mitochondrial OXPHOS and ATP generation, and, therefore, improve chemoresistance (Marlein et al., 2017; Shafat et al., 2017; Marlein et al., 2018). Inhibition of electron transport complex I by metformin reduces mitochondrial transfer from BM-MSC to AML, resulting in enhanced sensitivity of AML cells to cytarabine treatment (You et al., 2022), while IACS-010759, a small complex I inhibitor, enhances mitochondrial trafficking from BM-MSC, triggers compensatory adaptation of leukemia cells to energetic stress via endogenous mitochondrial fission and mitophagy, and facilitates AML resistance (Saito et al., 2021). As leukemia-initiating AML cells can reside in the CD34^+^CD38^+^ compartment, treatment with the recombinant antibody daratumumab against CD38 inhibits mitochondrial transfer to AML blasts, inhibiting the leukemia growth (Mistry et al., 2019). ATRA treatment has been shown to increase the CD38 expression in multiple myeloma, where it helps in mitochondrial transfer from BM-MSCs to myeloma cells (Marlein et al., 2019). Extracellular vesicles (EVs) released by MSCs have also been demonstrated to transfer mitochondria to neighboring cells, albeit the precise role of EV-mediated mitochondrial transport in leukemia remains unclear (Phinney et al., 2015; Griessinger et al., 2017) and necessitates more investigation to determine the precise mechanism for which BM-MSC mitotransfer may be involved in elimination of minimal residual leukemia.

## 5 Concluding remarks

BM-MSCs represent a powerful tool due to their regenerative potential and their immunomodulatory characteristics. Although challenges are associated with MSC isolation, limited *ex vivo* expansion, short life after transplantation, and the mode of *in vivo* administration must be addressed in order to improve hematopoietic outcomes. Metabolic coupling between BM-MSCs and HSCs is a crucial mechanism of fine-tuning of regeneration hematopoiesis, frequently hijacked by hematological malignancies. Coupling is highly dependent on the metabolic state of both cell compartments, and a major mediator of such metabolic coupling resides in their ability to exchange mitochondria and potentially other organelles. Identification of the molecular signals controlling the mitochondria exchange and overall metabolic coupling opens significant vulnerability opportunities for intervention and therapy in hematological malignancies.
